# When It Comes to an End: Oxidative Stress Crosstalk with Protein Aggregation and Neuroinflammation Induce Neurodegeneration

**DOI:** 10.3390/antiox9080740

**Published:** 2020-08-12

**Authors:** Patrycja Michalska, Rafael León

**Affiliations:** 1 Instituto Teófilo Hernando y Departamento de Farmacología y Terapéutica, Facultad de Medicina, Universidad Autónoma de Madrid, 28029 Madrid, Spain; 2 Instituto de Investigación Sanitaria, Servicio de Farmacología Clínica, Hospital Universitario de la Princesa, 28006 Madrid, Spain; 3 Instituto de Química Médica, Consejo Superior de Investigaciones Científicas (IQM-CSIC), 28006 Madrid, Spain

**Keywords:** neurodegenerative diseases, oxidative stress, protein aggregates, neuroinflammation, Nrf2-ARE pathway, reactive species, redox signaling

## Abstract

Neurodegenerative diseases are characterized by a progressive loss of neurons in the brain or spinal cord that leads to a loss of function of the affected areas. The lack of effective treatments and the ever-increasing life expectancy is raising the number of individuals affected, having a tremendous social and economic impact. The brain is particularly vulnerable to oxidative damage given the high energy demand, low levels of antioxidant defenses, and high levels of metal ions. Driven by age-related changes, neurodegeneration is characterized by increased oxidative stress leading to irreversible neuronal damage, followed by cell death. Nevertheless, neurodegenerative diseases are known as complex pathologies where several mechanisms drive neuronal death. Herein we discuss the interplay among oxidative stress, proteinopathy, and neuroinflammation at the early stages of neurodegenerative diseases. Finally, we discuss the use of the Nrf2-ARE pathway as a potential therapeutic strategy based on these molecular mechanisms to develop transformative medicines.

## 1. Introduction: Neurodegenerative Diseases

Neurodegenerative diseases (NDDs) are a heterogeneous group of neurological disorders, characterized by a progressive loss of particular subsets of neurons in different functional anatomic systems of the brain or the spinal cord [1]. NDDs include Alzheimer’s disease (AD), Parkinson’s disease (PD), and Amyotrophic Lateral Sclerosis (ALS), among others.

Given the increase in life expectancy and the lack of effective treatments for NDDs, the already large number of affected individuals is expected to increase enormously. During the last decades, tremendous efforts have been made to find effective disease-modifying treatments for these devastating diseases; however, the lack of efficient diagnosis and the still unknown causes of NDDs have hampered the development of successful therapies. Current treatment options are mainly symptomatic, condemning these patients to progressive deterioration, and eventually, to death.

NDDs pathophysiology represents a complex network of pathological events where several factors participate in their onset and development. Increasing evidence demonstrates that NDDs share many common pathophysiological mechanisms [2,3,4]. Among them, oxidative stress (OS), mitochondrial dysfunction, metal ions accumulation, neuroinflammation, protein misfolding, cell–cell transmission of characteristic disease-related proteins and apoptotic neuronal death have been widely described.

Numerous genetic mutations are associated with the onset of NDDs [5]; however, these “familial forms” represent less than 5% of the cases, and therefore, the vast majority of the cases are sporadic. It is known that environmental factors, lifestyle, or vascular risk factors influence the apparition of NDDs [6], but still, the major known risk factor for most NDDs is aging. OS has been widely recognized as an important factor in the etiology of NDDs [7,8], yet its role as a cause or consequence is debated. Herein, we will define the role of OS and its connection with NDDs. Not only evidence of OS in NDDs will be highlighted; we will also address how this ubiquitous process triggers selective neuronal vulnerability. Importantly, the interplay between OS and major features of NDDs, cellular proteostasis, and neuroinflammation, as key drivers of neurodegeneration will be addressed. Finally, therapeutic strategies based on these common pathological mechanisms will be discussed.

## 2. Reactive Species and Oxidative Stress

**Figure 1:**
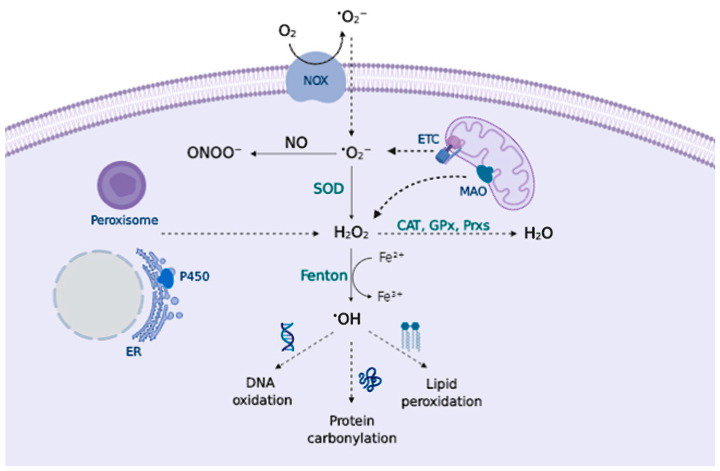
Principal RS sources and sinks. Transmembrane NADPH oxidases (NOX) and the mitochondrial ETC generate O_2_^•−^ that can be converted to ONOO^−^ in presence of NO, or can be converted to H_2_O_2_ by superoxide dismutase (SOD). Peroxisomes, endoplasmic reticulum (ER), and mitochondrial monoamine oxidase (MAO) also contribute to H_2_O_2_ production. Catalase (CAT), glutathione peroxidase (GPx), and peroxiredoxin (Prx) remove H_2_O_2_; otherwise, metal-catalyzed Fenton reactions generate •OH that oxidizes cellular biomolecules.

Reactive species (RS) have gained attention in the biological context given their role in cellular signaling and disease [9]. They are generally categorized as Reactive Oxygen Species (ROS) and Reactive Nitrogen Species (RNS). The most relevant RS include: superoxide anion (O_2_^•−^); hydroxyl radical (•OH); nitric oxide (NO); and non-radicals: hydrogen peroxide (H_2_O_2_); peroxynitrite (ONOO^−^) [10,11] (Figure 1).

In aerobic organisms, cellular metabolism is followed by oxygen-derived radical accumulation. RS play key physiological roles, being critical for cellular signaling and pro-survival pathways [12]; however, any disruption of the antioxidant/pro-oxidant balance in favor of the former leads to excessive RS generation called OS, causing toxic effects through irreversible damage to different biomolecules and alteration of different signaling pathways. While RS can be generated from several sources, numerous redox-sensitive targets and antioxidant systems control their cellular availability [13].

### 2.1. Sources of Reactive Species

Major sites of RS production are enzymatic endogenous sources, primarily the mitochondrial electron transport chain (ETC) [14] and the transmembrane NADPH oxidase (NOX) system [15], that form the superoxide anion (O_2_^•−^), which dismutates to form H_2_O_2_ and can be reduced to the hydroxyl radical (•OH) in metal-catalyzed Fenton reactions. O_2_^•−^ can also react with other radicals, notably nitric oxide (NO), producing peroxynitrite (ONOO^−^) (Figure 1). Complex I and III of the ETC are responsible for superoxide production in the mitochondria via a non-enzymatic process, hence, at a higher rate of metabolism, O_2_^•−^ production increases proportionally [16]. In fact, it is estimated that around 1–2% of the oxygen consumed reacts with electrons to form the superoxide anion [17]. Moreover, NOX occupy different cellular localizations and contribute to the local generation of O_2_^•−^.

There are other endogenous sources that contribute to RS production, e.g., peroxisomes [18]; the cytochrome P450 enzymes produce RS at the endoplasmic reticulum (ER) [19] or the mitochondrial enzyme monoamine oxidase (MAO) that catalyzes the oxidative deamination of biogenic amines, contribute to H_2_O_2_ production within the cells [20]. Cyclooxygenases (COX) and lipoxygenases (LOX) generate lipid-derived RS, such as prostaglandins [21] and leukotrienes [22], that are important mediators of inflammation. Lastly, exposure to environmental factors contributes to RS generation. Exogenous RS sources include drugs, heavy metals, pollutants, toxicants as well as UV and other ionizing radiation [23].

### 2.2. Biology of Reactive Species

Under physiological conditions, RS play key roles on critical pathways within the cell acting as signaling molecules in cell survival and differentiation, cell adhesion, immune responses, regulation of vascular tone, synaptic plasticity, and programmed cell death, among others [12,24]. When RS production becomes dysregulated, these oxidation processes may lead to permanent damage, followed by cell death. Considering the dual role of RS, their cellular concentration must be tightly regulated in order to maintain redox homeostasis, hence, cells possess different antioxidant mechanisms.

Superoxide anion (O_2_^•−^) is formed by a one-electron reduction of molecular oxygen. This process widely occurs via enzymatic reaction, catalyzed by NOXs [25,26] and xanthine oxidases [27]; via non-enzymatic electron transfer reactions such as the semiquinone in the mitochondrial ETC [28]; or via “auto-oxidation” reactions of non-radical compounds catalyzed by transition metals; e.g., dopamine (DA) or cysteine (Cys) [29]. The major physiological role attributed to superoxide is to kill foreign organisms in phagocytic cells, known as the respiratory burst of the immune system [30,31]. In addition to pathogen defense, superoxide may also control cell growth and vasoconstriction [32]. Increased levels of O_2_^•−^ can damage proteins containing iron-sulfur clusters [33] releasing free iron (Fe^2+^), or react with molecules such as catecholamines to deactivate them [34]. To prevent the superoxide toxic effect, aerobic organisms possess different isoforms of superoxide scavenging enzymes, superoxide dismutase (SOD), that catalyze superoxide dismutation into H_2_O_2_ and molecular oxygen [35] (Figure 1).

**Figure 2:**
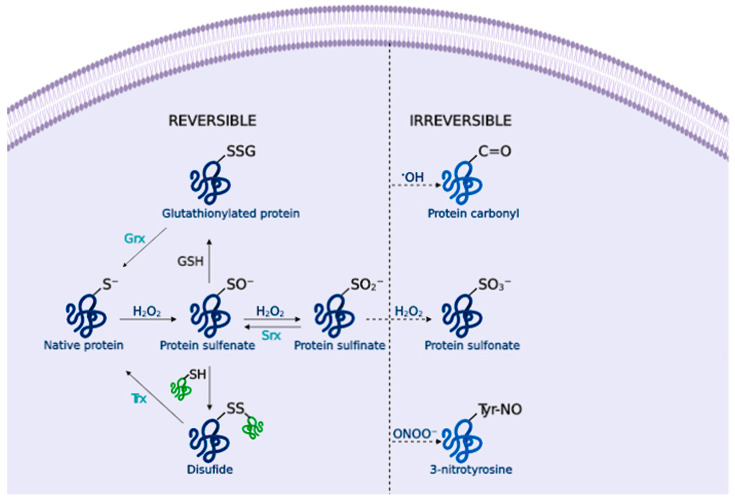
Cysteine redox modification by H_2_O_2_. Low H_2_O_2_ concentrations lead to sulfenates (SO^−^) formation that can form disulfide (SS) or glutathionylated proteins (SSG), and sulfinates (SO_2_^−^). These modifications can be reversed by thioredoxins (Trx), glutaredoxins (Grx) or sulfiredoxins (Srx). Under OS, the formation of sulfonate (SO_3_^−^) leads to irreversible damage. Likewise, amino acid oxidation by •OH or ONOO^−^ forms protein carbonyls and 3-nitrotyrosine residues that alter the protein function.

Hydrogen peroxide (H_2_O_2_) is an important redox signaling molecule [36]. Produced by SOD from superoxide, at physiological concentrations, H_2_O_2_ is capable of reversible oxidation of proteins, triggering different cellular processes such as proliferation, migration, differentiation, or angiogenesis [36]. H_2_O_2_ intracellular concentrations can be reduced by the action of catalase (CAT), peroxiredoxin (Prx), or glutathione peroxidase (GPx) [37] (Figure 1); otherwise, H_2_O_2_ redox-mediated signaling predominantly involves the reaction with thiolate (S-) from cysteine (Cys) residues to form a sulfenate (SO^−^) that can form intra- or inter-molecular disulfide bonds (SS) in the presence of other thiols, thus inducing protein modifications that alter its function. [38] These oxidized forms can be reversed by thioredoxins (Trx) or glutaredoxins (Grx), ensuring a reversible signal transduction mechanism. Sulfenate can be further oxidized to sulfinate (SO_2_^−^), which can be reduced by sulfiredoxin (Srx) [39]; however, excessive H_2_O_2_ concentrations further oxidize these proteins to sulfonate (SO_3_^−^), leading to irreversible oxidation of biomolecules and H_2_O_2_-mediated toxicity [38] (Figure 2).

Hydroxyl radical (•OH) is formed from H_2_O_2_ by metal-catalyzed (Fe^2+^ or Cu^+^) Fenton reactions [40]. Additionally, superoxide can interact with H_2_O_2_ to undergo a Haber-Weiss reaction catalyzed by a metal ion to form •OH [41]. •OH is extremely reactive, thus it reacts close to its formation site, and indiscriminately oxidizes lipids, proteins and DNA, causing severe cellular damage [42] (Figure 1). For example, •OH is able to directly oxidize amino acid side chains (via metal-catalyzed reactions), or cause protein backbone cleavage, leading to the irreversible formation of carbonyl groups [43] (Figure 2).

Nitric oxide (NO) is endogenously synthesized by nitric oxide synthases (NOS) from L-arginine in endothelial cells, neurons, and macrophages, among others. NO is important for several physiological processes, including neurotransmission, synaptic plasticity in the central nervous system (CNS), and immune regulation [44]. RNS overproduction may lead to nitrosylation reactions on proteins, altering their structure and function [45]. NO toxicity greatly increases upon reaction with O_2_^•−^, as it produces peroxynitrite (ONOO^−^) [46], a potent pro-inflammatory and cytotoxic molecule [47,48]. ONOO^−^ is highly reactive and can oxidize lipids [49], DNA [50], and methionine and tyrosine protein residues [51] (Figure 2). Besides, ONOO^−^ is able to nitrate SOD, thus promoting O_2_^•−^ accumulation and damage [52].

### 2.3. Signalling Pathways and Oxidative Stress

In addition to the aforementioned functions, RS are involved in a plethora of signal transduction pathways. They play important roles as secondary messengers in cell growth, differentiation, and proliferation [53,54]. Supporting this observation, there is a large number of redox-sensitive proteins in cells [55]. Growth factors bound to tyrosine kinase receptors increase intracellular RS that are essential for downstream signaling events [56]. H_2_O_2_ can target Cys residues at the active sites of protein tyrosine phosphatases [57], playing an important role in the modulation of mitogen-activated protein kinases (MAPKs) signaling pathways. As a consequence, different pathways are activated, such as the phosphatidylinositol 3-kinase (PI3K)/Akt pathway [58], the extracellular-regulated kinases (ERKs), the c-Jun N-terminal kinases (JNKs), or the p38-MAPK cellular pathways [12].

In addition to the activation of signaling pathways, cells have several mechanisms to face changes in RS concentration. This involves activation of nuclear transcription factors, such as the activator protein 1 (AP-1) transcription factor, important for cell growth and differentiation [59], or the hypoxia-inducible factors (HIFs), responsible for the cell adaptation to low oxygen; both factors are degraded in a ROS-dependent manner [60].

A primary defense cellular mechanism against OS is the antioxidant Nrf2-ARE pathway that regulates the expression of the phase II antioxidant response enzymes [61,62]. Under normal conditions, Nrf2 is sequestered in the cytosol by Keap1 (a Kelch-like erythroid cell-derived protein with CNC homology (ECH)-associated protein 1), which is an adaptor protein for a Cullin 3 (Cul3) scaffold protein of Nrf2 ubiquitin ligase (E3) that promotes Nrf2 ubiquitination and degradation via the proteasome [63]. In the presence of exacerbated OS, key Cys residues at Keap1 are selectively oxidized [64,65], leading to a conformational change in Keap1 that prevents Nrf2 ubiquitination. Then, Nrf2 cytosolic concentration increases and it is translocated to the nucleus, where it binds to the ARE sequences promoting the expression of phase II enzymes. Moreover, the Nrf2-ARE pathway indirectly modulates other pathways, through crosstalk, like the Nuclear factor-kappa B (NF-κB) pathway.

Among the antioxidant enzymes regulated by the Nrf2-ARE pathway, SODs catalyze O_2_^•−^ conversion to H_2_O_2_, which can be further removed by the action of GPx. PRXs are thiol-dependent peroxidases that catalyze the reduction of H_2_O_2_, organic hydroperoxides, and peroxynitrite [66] (Figure 1). Heme oxygenase-1 (HO-1), responsible for the degradation of the pro-oxidant heme group, releasing carbon monoxide (CO), biliverdin, and free iron.

Amongst non-enzymatic antioxidant systems, glutathione (GSH) is the most important antioxidant and redox buffer of the cell. Nrf2 target genes are involved in the synthesis of GSH: glutamate-cysteine ligase (GCL), glutathione reductase (GR) and glutathione S-transferase (GST). GSH can act as a direct antioxidant (by reaction with O_2_^•−^ and •OH), transport and store cysteines, and it acts as an electron donor and maintains the redox potential in cells [67] (Figure 2).

NF-κB is another pro-survival transcription factor activated by ROS that regulates several cellular defense mechanisms, such as cellular survival (transcription of anti-apoptotic proteins and inhibition of caspase-dependent cell death pathways), growth, and differentiation, and it is a master regulator of inflammation [68]. Under normal conditions, NF-κB is repressed at the cytosol by its inhibitor (IκB) and it is subjected to a complex regulation [69]. RS regulation of the NF-κB pathway has different outcomes depending on the cellular localization. In the cytoplasm, ROS promote IκB phosphorylation and subsequent NF-κB translocation to the nucleus to activate the proinflammatory signaling pathways; in contrast, RS may inactivate NF-κB by inhibiting its binding to DNA in the nucleus [70].

### 2.4. Oxidative Stress-Driven Neurodegeneration

The progression of age-related NDDs decreases the ability of cells to maintain redox homeostasis, promoting the accumulation of RS and neuronal injury. In addition to oxidative damage to biomolecules, RS toxicity in neurons contributes also to mitochondrial dysfunction [71], glial cells activation, protein misfolding and aggregation, leading, finally, to cellular apoptosis [72]; hence, OS damage promotes neurodegeneration. Low levels of RS enhance selective removal of damaged mitochondria by mitophagy [73], while high levels of RS can trigger mitochondrial permeability transition pore (mPTP) opening, leading to mitochondrial depolarization, loss of ATP production and Ca^2+^ levels elevation [74]; thus, OS contributes to cellular apoptosis, necrotic cell death, and ferroptosis [75] in NDDs [76]. RS can also activate matrix metalloproteinases, contributing to the blood-brain barrier (BBB) disruption [77], facilitating neurotoxic blood-derived products, and entry of peripheral inflammatory cells.

Oxidative damage occurs in all aerobic cells, yet the brain is particularly susceptible to OS [78]. Firstly, the brain is a highly energy-demanding tissue. It consumes about 20% of total oxygen, necessary to generate ATP to maintain all the openings and closings of ion channels associated with the propagation of action potentials and neurosecretion [29]. Other factors that contribute to brain vulnerability are:

- Mitochondrial DNA accumulates mutations during aging, therefore, mitochondria become less efficient in producing energy, and more efficient in generating free radicals. Neurons are highly dependent on oxidative phosphorylation, compared to other cells, and thus they become more sensitive to OS [7].
- Mitochondrial failure leads to a drop in ATP content, thus, other energy sources might become important, such as aerobic glycolysis or the mitochondrial tricarboxylic acid cycle. However, these energy-producing systems have been demonstrated to be dysregulated in NDDs [79,80].
- Several neurotransmitters are auto-oxidizable. DA, L-DOPA, or norepinephrine can react with O_2_ to generate O_2_^•−^ and quinones/semiquinones that can deplete GSH and react with protein Cys residues [81].
- High levels of iron are found throughout the brain, where iron serves as an essential cofactor for proteins involved in normal neuronal function (cytochrome P450, ferritin, components of the mitochondrial ETC); nevertheless, iron can catalyze free radical formation via a Fenton reaction causing damage [82].
- Neuronal membrane lipids, rich in polyunsaturated fatty acids, can readily undergo lipid peroxidation. Furthermore, peroxidation products are highly neurotoxic, e.g., 4-hydroxynonenal (HNE) can trigger mPTP opening, increase free Ca^2+^ levels, and favor glutamate toxicity [83,84].
- MAO enzymes in the brain catalyze the oxidative deamination of biogenic amines, greatly increasing H_2_O_2_ content [20].
- Antioxidant defenses are relatively low and decrease during aging, as demonstrated for GSH content [85].

In addition to intrinsic brain vulnerability to OS, certain neuronal cells are particularly prone to oxidative damage, known as selective neuronal vulnerability [86,87]. As such, pyramidal, cholinergic and noradrenergic neurons from the hippocampal CA1 region [88], entorhinal cortex [89], locus coeruleus [90] and basal forebrain [91] are vulnerable to neurodegeneration in AD; PD is characterized by neurodegeneration of dopaminergic neurons in the *substantia nigra* pars compacta (SNpc) [92]; while motor neurons in the motor cortex and spinal cord primarily undergo cell death in ALS [93]. Different mechanisms have been proposed as factors responsible for the selective neuronal vulnerability, including: (i) elevation of Ca^2+^ cellular levels make neurons expressing calcium-buffering proteins less vulnerable than those lacking these proteins [94,95]; (ii) perturbation of energy demand and mitochondrial activity makes large axonal arbors more susceptible, as in the case of dopaminergic neurons in the (SNpc) [96]; (iii) different expression patterns of glutamate receptors make neurons more vulnerable or resistant to degeneration [97,98]. Other mechanisms, such as higher neuronal vulnerability to proteostasis alterations, or the increased expression of neurofilaments, which tend to aggregate in large neurons, have been described. However, precise mechanisms of neuronal vulnerability are yet to be defined [1,87].

### 2.5. OS Induced Degeneration in AD, PD and ALS

Different studies have demonstrated that oxidative damage is increased in AD patients compared to elderly controls at the early stages of the disease [99]. Amyloid beta (Aβ) and hyperphosphorylated-tau (P-tau) accumulation in AD increases OS, leading to mitochondrial dysfunction [100]. Aβ plaques bind Cu^2+^ and Fe^3+^ ions, facilitating H_2_O_2_ decomposition and •OH production [101]. In turn, OS might increase Aβ production and aggregation and facilitates tau phosphorylation and polymerization, thus forming a vicious cycle that promotes the initiation and progression of AD.

OS is also a major pathophysiological mechanism in PD. The fact that mitochondrial complex I inhibitors such as 1-methyl-4-phenyl-1,2,3,6-tetrahydropyridine (MPTP) cause clinical parkinsonism reveals a close relationship between mitochondrial dysfunction and PD. The reduced activity of complex I in the SNpc of PD patients has been linked to excessive RS production and subsequent apoptosis [102,103]. In addition, genes responsible for familial PD affect mitochondrial function, increasing OS [104]. Other pieces of evidence are related to the finding of the depletion of GSH levels in the SNpc at the early stages of PD, and increased iron levels [105].

In familial ALS, 20% of the cases result from mutations in SOD1, leading to a toxic gain of function of the enzyme as it loses the active sites for Cu^2+^ that converts the enzyme into a pro-oxidant protein that participates in RS generation [106]. SOD1 mutations increase NOX2-dependent RS production, which may cause motor neuron death [107].

## 3. Proteinopathy in NDDs

**Figure 3:**
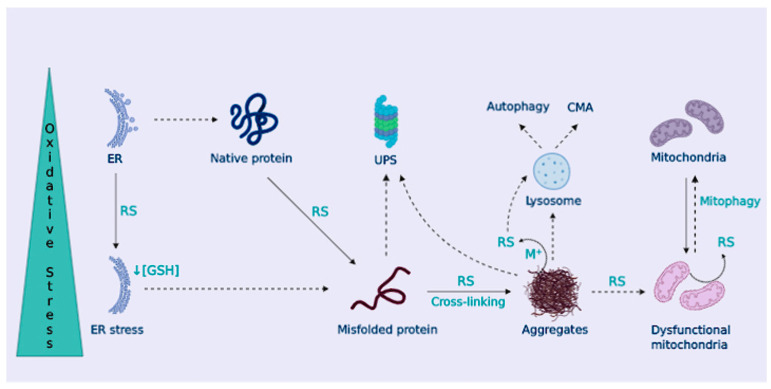
Cellular proteostasis is lost under OS. OS promotes protein misfolding and hydrophobic core exposure, leading to the formation of protein aggregates. Protein control systems, ubiquitin-proteasome system (UPS), chaperone-mediated autophagy (CMA), and macroautophagy (autophagy) are harnessed by OS, favoring protein aggregation. In addition, protein aggregates trap metal ions to produce RS-impairing mitophagy, leading to the accumulation of dysfunctional mitochondria. Also, the ER is damaged by OS, further promoting protein misfolding.

The presence of characteristic aberrant protein aggregates, as a consequence of proteostasis deregulation, is a relevant aspect in NDDs pathophysiology [108,109]. Misfolded proteins are targeted for ubiquitin proteasome system (UPS) degradation, or chaperone-mediated autophagy (CMA) degradation when tagged with a KFERQ motif; however, when UPS and CMA capacity is overwhelmed, aggregation-prone proteins form inclusion bodies that are delivered to the macroautophagy system (generally known as autophagy) (Figure 3).

Aberrant protein elimination is highly sensitive for neurons due to their inability to divide (and, therefore, to reduce their toxic aberrant aggregates content); in addition, protein aggregates need to be packaged in axons and dendrites to finally undergo a retrograde journey to the soma that is rich in lysosomes. During aging, molecular components and activity of UPS, CMA, and autophagy are down-regulated [110,111,112]. In addition, in many NDDs, misfolded proteins tend to aggregate in a β-sheet conformation that becomes neurotoxic, and they are able to propagate and further damage the activity of proteolytic systems.

### OS Contributes to Dampen Cellular Proteostasis

OS contributes to protein misfolding and aggregation by several means. Proteasomal or lysosomal removal of damaged proteins may be altered by OS [113]. In turn, the accumulation of misfolded proteins leads to the formation of oligomers and higher aggregates that can interact with metal ions and catalyze RS production, as already described in NDDs [114]. Moreover, the formation of protein aggregates can impair mitochondrial bioenergetics or alter the removal of dysfunctional mitochondria through mitophagy [115,116], leading to their accumulation, which further enhances RS generation and neuronal death (Figure 3). Characteristic protein aggregates of NDDs can also disrupt the mitochondria-ER association, leading to disrupted Ca^2+^ homeostasis and lipid metabolism, among others [117]. The ER is in charge of proper protein folding. OS disrupts ER function causing protein misfolding and ER stress that leads to the activation of the unfolded protein response (UPR) [118]. Also, given the high rate of disulfide bond formation during protein folding in the ER, ER stress contributes to OS by depleting GSH content [119]. In NDDs, activation of the UPR has been widely described, suggesting a role for ER stress in neurodegeneration [120] (Figure 3).

OS also influences protein function and folding by several pathways as a result of the direct reaction of RS with them. As such, NO can form RNS that can nitrate protein tyrosine residues, modifying the protein structure and function [121]. Similarly, H_2_O_2_ mediates redox signaling via reversible protein oxidation [36]. Cys residues are prone to RS oxidation because they possess highly nucleophilic thiol groups, thus, several cellular antioxidant systems act as redox switches to preserve the protein pools [38]. In contrast, there are no systems that can reverse protein carbonylation (•OH oxidation), thus, protein carbonylation is normally used as an OS marker [122] (Figure 2). Protein carbonylation (at proline, threonine, lysine, and arginine residues [123]) leads to protein unfolding and exposure of the hydrophobic core, and consequently, decreased solubility. Oxidized proteins tend to interact to form insoluble protein aggregates [124] commonly observed in NDDs. Moreover, side reactions between a carbonyl group from a carbonylated protein with an amino acid from a second protein forms a Schiff-base that further contributes to protein aggregation [125]. Additional cross-linking reactions mediated by RS, like lipid peroxidation products, may take place, amplifying the aggregation processes [126].

The formation of aggregates composed of heavily oxidized proteins can inhibit proteasome activity [126], which may be responsible for the down-regulation of proteasomal activity observed during aging [126]. In this sense, it has been previously reported that there is a decline in the proteasome 26S subunit activity caused by OS [127]. Other UPS components can also be altered by OS, e.g., ubiquitin ligases can be modified by S-glutathionylation [128] or S-nitrosylation [129], modifying their clearance capacity (Figure 3). OS can oxidize proteins, and this can promote damage to the proteasome, likewise, OS directly harness the proteasome system. Altogether, these alterations promote accumulation and aggregation of proteins, spreading the advance of NDDs [130,131].

In the context of NDDs, the removal of aggregated proteins via autophagy is critical [132]. Autophagy deficits are likely to contribute to neurodegeneration in various diseases [133] and enhance the aggregation of pathological proteins (Aβ peptide and Tau in AD, α-synuclein in PD, and SOD1 and TAR DNA-binding protein 43 (TDP-43) in ALS). Basal redox-signaling up-regulates autophagy flux [134], indicating a pro-survival response to eliminate oxidized proteins and prevent OS-induced apoptosis, while excessive activation of autophagy promotes cellular death. Redox-sensitive autophagy proteins like p62 possess oxidizable Cys residues that increase autophagy flux upon oxidation [135]. Similarly, OS conditions intensify the removal of oxidized proteins through CMA by upregulating LAMP-2A levels [136]. OS can also destabilize lysosomes, impairing autophagy [137], which leads to the accumulation of cross-linked aggregates in the form of lipofuscin [138] (Figure 3). Nonetheless, the effect of chronic OS and impaired autophagy in NDDs still needs further investigation [139].

## 4. Neuroinflammation in Neurodegeneration

The immune response within the brain is a tightly regulated process constituted by different molecular and cellular mechanisms including immune cells, blood vessels, and molecular mediators [140]. Microglia and astrocytes, and also, peripherally derived T cells, macrophages, and dendritic cells inspect the healthy CNS for harmful agents [141].

**Figure 4:**
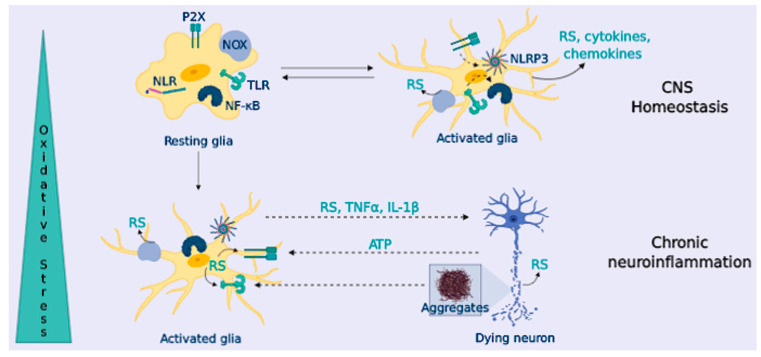
OS triggers chronic neuroinflammation, promoting neurodegeneration. Glial cells express TLR and NLR that sense harmful agents. Glial activation through TLR triggers the NF-κB signaling pathway and NLR like ligand-gated ionotropic purinergic receptor (P2X) that triggers NLRP3 signaling, promoting cytokine release, and also chemokines and RS (from NOX and iNOS enzymes). In healthy CNS, glial cells resolve the inflammatory response; in NDDs, the presence of protein aggregates and factors released from dying neurons leads to a sustained glial activation that contributes to neuronal demise.

To provide innate immunity, microglial cells express Toll-Like Receptor (TLRs), NOD-like receptors (NLRs), and scavenger receptors [142]. When microglial cells are triggered (“classical activation”), they release high levels of pro-inflammatory cytokines such as tumor necrosis factor α (TNFα), interleukin 1 beta (IL-1β) and IL-6, and chemokines (such as monocyte chemoattractant protein-1, MCP-1), and they increase the expression of the inducible nitric oxide synthase (iNOS) and NOX enzymes [143] (Figure 4). Following classical activation, microglia switches to an “alternative activation” that participates in inflammation resolution and tissue repair. These microglial cells are characterized by branched processes, and they release anti-inflammatory cytokines (IL-10 or IL-4), neurotrophic factors, and up-regulate molecular markers such as Arginase-1 and transforming growth factor beta (TGFβ) [144].

Astrocytes are important for the formation of neuronal synapses, energy and metabolic support, and regulation of BBB permeability [145]. In the injured CNS, astrocytes become reactive, migrate to the damaged site, and form the glial scar [146]. Astrocytes also respond to inflammatory stimuli in the CNS. They express TLRs and scavenger receptors in their membrane, and they release pro-inflammatory mediators when stimulated with cytokines released by microglia [142,147]. Neuronal regions with a lower density of astrocytes are more susceptible to oxidative damage, as astrocytes are thought to clear-off RS in the environment [148].

### OS and Neuroinflammation

RS are key second messengers in the innate and adaptive immune response, however, RS overproduction may lead to a sustained activation of the inflammatory response in immune cells, causing tissue damage and pathology [149] (Figure 4). In the innate immune response, mitochondria-derived RS can act as signaling molecules to up-regulate pro-inflammatory cytokine production [150]; in turn, these cytokines can cause RS up-regulation upon activation of iNOS (hence, NO excess) and NOX (O_2_^•−^). Besides, RS are important for the activation of PRRs signaling pathways, [151]; likewise, NOX-derived and mitochondrial RS can activate the inflammasome component nucleotide-binding domain leucine-rich repeat and the pyrin domain containing receptor 3 (NLRP3) [152], increasing the production of IL-1β (Figure 4).

In addition to the microglial-astrocyte crosstalk, neurons also participate in the immune response. Neurons sense glial-released pro-inflammatory factors; in response, they can release inhibitory factors to resolve inflammation, or they can further activate glial PRRs receptors through the release of damage-associated molecular patterns (DAMPs), such as ATP or DNA [153] (Figure 4). In addition to the microglial-astrocyte crosstalk, neurons also participate in the immune response. Neurons sense glial-released pro-inflammatory factors; in response, they can release inhibitory factors to resolve inflammation, or they can further activate glial PRRs receptors through the release of damage-associated molecular patterns (DAMPs), such as ATP or DNA [153] (Figure 4). In fact, neuron-glial crosstalk is necessary for the correct function of glial cells and neurons, significantly contributing to brain homeostasis [154]. Communication between glial cells and neurons is mediated by a plethora of signals and receptors, including neurotransmitters (glutamate, GABA, or serotonin) and their receptors. It also includes the purinergic and adenosine signaling of different chemokines including fractalkine (a chemokine produced by neurons that signals directly in microglia) [155]. The complement system is also present in neurons and glia, and this communication is reported to be altered in NDDs [156]. The interconnection between neurons and the immune system has been concisely reviewed by Marinelli et al. [154].

Under some circumstances, glial cells undergo an uncontrolled activation, leading to chronic neuroinflammation that triggers an exaggerated inflammatory response, the release of neurotoxic factors and, finally, to neuronal loss (e.g., chronic exposure to TNFα can cause neuronal death) [157] (Figure 4). Given the limited microglia replication and turnover, microglial cells (and astrocytes) change to a more inflammatory phenotype, known as microglial priming, during aging [158]. In NDDs, there is increased microglial priming that enhances the neuro-inflammatory-derived toxicity and boosts neurodegeneration [159]. In addition, intrinsic characteristics of NDDs, like OS and the presence of protein aggregates, lead to a sustained microglial activation that becomes deleterious.

## 5. Pathological Crosstalk in Neurodegeneration

In previous sections, we have addressed the harmful role of OS, protein aggregation, and neuroinflammation in NDDs. However, it is necessary to highlight the interconnection among these factors as key drivers of neurodegeneration at the early stages of disease development.

(I) OS is responsible for the oxidative damage to cell biomolecules and RS are essential to the initiation of immune responses. Also, RS activate redox-sensitive transcription factors such as NF-κB, crucial for the neuroinflammatory response. Chronic NF-κB activation leads to sustained microglial activation [160] that increases OS. OS can compromise BBB integrity, favoring the infiltration of peripheral immune cells, contributing to neuroinflammation and neurodegeneration. Protein oxidation by carbonylation cannot be reversed and it leads to protein unfolding, hydrophobic residue exposure, and decreased solubility, thus enhancing the risk for protein aggregation. Furthermore, additional cross-linking reactions favor the formation of protein aggregates and further extend oxidative damage [125]. Various cellular systems related to the removal of misfolded proteins and protein aggregates that control cellular proteostasis are dysregulated. OS can destabilize lysosomes, impairing autophagy [137] through Fenton reactions that lead to lysosomal iron-mediated rupture, and can also alter the UPS (Figure 3).

(II) In NDDs characterized by the presence of aberrant protein aggregates, these aggregates can become RS sources themselves as they tend to coordinate metal ions, increasing radical production via Fenton chemistry. Moreover, aberrant protein aggregates act as DAMPs signals on microglial cells, triggering the inflammatory response [161]. In NDDS, the proteolytic systems become deficient, and misfolded proteins continue to aggregate, promoting a sustained microglial activation that leads to chronic neuroinflammation.

(III) Glial inflammatory activation contributes to OS by increasing the expression of iNOS and NOX enzymes and promotes endoplasmic reticulum stress that aggravates protein misfolding [162]. Compelling pieces of evidences have demonstrated that the release of cytokines, chemokines, and RS from activated glial cells harnesses autophagy when it becomes sustained [163]. In addition to the relation with OS, neuroinflammation further enhances neurodegeneration by altering cellular proteostasis, and it has been demonstrated that IL-1β and IL-6 can block autophagy in microglia [164]. Consequently, chronic neuroinflammation leads to autophagy failure, increasing the accumulation of protein aggregates, that, in turn, activate glial cells, initiating a positive feedback loop that contributes to neurodegeneration. Finally, these factors induce neuronal damage, and eventually, neuronal death. Dying neurons release different factors such as ATP that activate P2X microglial receptors triggering NLRP3 inflammasome, and they also release RS or glutamate contributing to excitotoxicity. Due to the cell-cell communication, feedback loops are established, spreading the damage that leads to neurodegeneration (Figure 4).

### 5.1. Alzheimer’s Disease

AD is a chronic, irreversible, and fatal NDD that affects 46.8 million people worldwide, being the most common cause of dementia during aging. AD is characterized by progressive cognitive impairment and memory loss and two major hallmarks, extracellular amyloid plaques, composed by aggregated Aβ peptide, and intracellular neurofibrillary tangles (NFTs), constituted by aberrant P-Tau [165]. Currently, AD is considered a multifactorial disease derived from a highly complex pathological network in which the initial causes remain elusive. Due to its complexity, the latest research hypotheses are questioning if these aggregates are a physiological response to pre-existing homeostasis deregulation that induces their production in response to a previous toxic insult. In this line, recent evidence points to a combination of pathological events at the early stages of the disease, and the interconnection among them, as potential effectors to initiate the complex cascade of events leading to AD development [166]. Therefore, in addition to protein aggregation, several pathological pathways are currently being investigated, including mitochondrial failure [167], OS [168], chronic neuroinflammation [169], and proteostasis dysregulation [170,171].

As previously described, OS is known to progressively increase during aging, being a key factor in the neuronal loss observed in AD as a consequence of mitochondrial dysfunction, metal dyshomeostasis, and defective antioxidant defenses [172]. Mitochondrial failure is an AD pathological hallmark and the main source of RS leading to the oxidation of protein, lipids, and nucleic acids. These oxidized products have been found in the post-mortem brains of AD patients [173] together with a decreased activity of mitochondrial cytochrome c oxidase [174], complex IV of the ETC. Interestingly, ROS and RNS overproduction are linked to Aβ production and toxicity and, in turn, Aβ increases RS, mitochondrial dysfunction, and cytosolic Ca^2+^ imbalance prior to amyloid plaques’ establishment [175]. These detrimental effects have been recently related to the Aβ ability to inhibit the cytochrome c oxidase [176], inducing energy depletion and mitochondrial failure. Furthermore, OS impairs the low-density lipoprotein receptor-related protein 1 (LRP1), a receptor involved in Aβ endocytosis for further clearance, oxidizing it and impairing its activity facilitating Aβ accumulation [177]. A 60% increase in oxidized LRP1 has been observed in the hippocampus of AD patients compared to age-matched controls. Furthermore, as previously depicted, Aβ oligomers and protofibrils are able to coordinate Cu^2+^, Zn^2+^ and Fe^2+^ metal ions increasing RS production via Fenton chemistry [114] and highly contributing to OS damage.

OS has also been related to tau hyperphosphorylation by a number of different pathways. OS increases the expression of regulator of Calcineurin 1 (RCAN1), the natural repressor of calcineurin (a phosphatase that dephosphorylates tau), augmenting tau phosphorylation [178]. Additionally, high RCAN1 levels increase glycogen synthase 3β (GSK-3β) expression, one of the most important kinases related to tau hyperphosphorylation, accelerating the formation of P-Tau and NFTs [179]. OS also accelerates P-Tau aggregation by the oxidation of a Cys residue on tau [180] or by nitration, S-nitrosylation and oxidation of methionine residues [181], increasing its aggregation and self-assembly capacity. Additionally, overactive GSK-3β facilitates Aβ formation and aggregation [182], induces OS, and participates in the activation of pro-inflammatory pathways [183]. A mayor role of GSK-3β on RS production in AD pathology is its ability to inhibit the Nrf2-ARE anti-oxidant pathway. GSK-3β phosphorylates Nrf2 at the Neh6 degron domain, creating a recognition site for β-TrCP, an E3 ligase adaptor that facilitates Nrf2 ubiquination and its proteasomal degradation [184]. Under pathological AD conditions, GSK-3β activity has been widely reported to be increased, thus, it decreases Nrf2-ARE pathway activation to exacerbate OS.

Chronic neuroinflammation plays a key role in AD onset and development. OS pathologically induces glial activation, mainly microgliosis and astrogliosis. Compelling evidence reports the apparition of increased levels of proinflammatory cytokines in the blood, cerebrospinal fluid (CSF), and post-mortem brain tissues of AD patients [185,186]. OS, oxidized biomolecules, and Aβ [187] are known to activate microglia, initiating the proinflammatory response. Once activated, microglia activate the expression different proinflammatory factors, including iNOS and NOX, that produce high amounts of RS and proinflammatory cytokines such as TNFα, and different proinflammatory interleukins (IL-1β or IL-18). In turn, NO liberated by iNOS is rapidly transformed into ONOO^−^ that readily nitrates Aβ, augmenting its self-aggregation capacity and toxicity [188]. Importantly, nitrated Aβ is the main component of amyloid plaques [189]. Aβ is able to activate the scavenger receptor CD36, stimulating sterile inflammation that promotes TLR4 and TLR6 activation to initiate the NF-κB proinflammatory pathway [190], further increasing OS and proinflammatory factors’ release. In turn, CD36 promotes Aβ oligomerization, acting as a priming signal for NLRP3 activation that liberates high amounts of IL-1β [191]. Finally, IL-1β increases p38-MAPK and GSK-3β activities, promoting tau hyperphosphorylation [192].

Considering proteostasis, the formation and accumulation of misfolded proteins have been associated with a dysregulation of these clearance pathways, including UPS [130], CMA, and autophagy processes [193,194]. Considering the UPS system, it was described that Aβ is able to inhibit this clearance system, promoting further Aβ and P-Tau accumulation [195]. Under OS conditions, essential proteins of the UPS are oxidized and lose their functions, such as the ubiquitin carboxy-terminal hydrolase L1 (UCHL1) [196,197], leading to increased levels of polyubiquitinated proteins, dysfunctional UPS, and accumulation of aberrant aggregates [198]. Moreover, OS can damage proteasome 26S, eliminating the two initial parts of the complex, preventing its capacity to process ubiquitinated proteins [130], and thus, accelerating the accumulation of abnormal proteins. Once accumulated, Aβ is prone to be processed by autophagy under OS pathological conditions [199]; however, its accumulation impairs the autophagosome-lysosome fusion, promoting the accumulation of non-processed vesicles at dystrophic neurites [200]. Additionally, hyperphosphorylated tau has been found to co-localize with LC3-II and p62 in AD patients, indicating a potential effort to eliminate these aberrant aggregates [201]. However, P-Tau also disrupts axonal transport, contributing to autophagosome accumulation in AD [202]. Furthermore, OS excessively inhibits autophagy via mTOR (mammalian target of rapamycin) [203,204]. Thus, the interconnection between OS, chronic neuroinflammation, and proteostasis failure can create a highly toxic environment for neurons [80], and, more importantly, the positive feedback loops generated accelerate the advance of the disease over time.

### 5.2. Parkinson’s Disease

PD is characterized by the progressive degeneration of dopaminergic neurons in the SNpc, as well as other brain regions, including the *locus coeruleus* and the *nucleus basalis* of Meynert [205]. The loss of dopaminergic signaling is responsible for the cardinal motor symptoms observed in the disease. The most prominent PD pathological hallmark is the presence of cytoplasmic inclusions composed of α-synuclein proteins, termed Lewy bodies [206]. Although PD etiology is not fully understood, environmental and/or genetic mutations, related to the familial forms, have been described [207,208]. From a molecular point of view, OS has been recognized as a principal mechanism leading to neuronal death in PD [209,210]. OS sources in PD include mitochondrial dysfunction, neuroinflammation, DA metabolism, and iron dysregulation; at the same time, mechanisms that control cellular homeostasis like the UPS and mitophagy are altered by OS and can, in turn, enhance RS production. The interplay among these factors generates a complex network that promotes neuronal demise.

Analysis of post-mortem brains shows that oxidative damage is present in PD, as revealed by the presence of HNE [211], protein carbonyls [212], and DNA oxidation products [213]. Dopaminergic neurons are prone to OS due to DA metabolism by MAO and auto-oxidation that produces RS within the cell. Likewise, DA auto-oxidation generates quinone intermediates that can deplete cellular GSH and modify PD related proteins such as α-synuclein, contributing to neurodegeneration [209]. In fact, reduced GSH levels are decreased in the SNpc of PD brains compared to age-matched controls [214]. GSH downregulation induces decreased mitochondrial complex I activity and higher neuronal vulnerability to OS, leading to nigrostriatal neurodegeneration [215]. Moreover, PD brains contain higher levels of iron in the SNpc compared to aged matched controls, contributing to the pathogenesis [216]. Lastly, dysfunction of mitochondrial complex I in the SNpc has been linked to PD pathology [103]; it can enhance mitochondrial DNA mutations, abnormal mitochondrial Ca^2+^ homeostasis, and OS, and it can cause α-synuclein aggregation [217]. Familial forms of PD caused by genetic mutations in DJ-1, PTEN-induced kinase 1 (PINK-1), parkin, and leucine-rich repeat kinase 2 (LRRK2) also impact the mitochondrial function, further evidencing the implication of OS in PD neurodegeneration.

The physiological role of α-synuclein remains elusive; to the date, it has been associated with synaptic function, neurotransmitter release, vesicle trafficking, and synaptic plasticity [218]. This presynaptic protein has a predisposition to form fibrillary structures and aggregates, eventually leading to cell death. The proteasome, CMA, and autophagy can degrade α-synuclein [219,220]; however, these systems are damaged in PD [221]. After OS damage, the proteasome impairment observed in PD [222] contributes to α-synuclein aggregation; in turn, aggregated α-synuclein further impairs proteasome activity [223].

Damaged CMA has also been linked to PD [224]. Interestingly, α-synuclein is modified by oxidized DA, blocking its CMA degradation. Modified α-synuclein also impairs the degradation of other substrates [225], suggesting a mechanism for selective dopaminergic neuronal degeneration in PD. Similar to α-synuclein, mutant forms of UCHL1 and LRRK2 interact with the lysosomal membrane receptor LAMP2A and block the CMA pathway [226,227]. α-synuclein accumulation can further harness the autophagy system [228]. Autophagy failure in PD is particularly important for mitochondrial control. Mitophagy is principally mediated by two proteins, PINK1 (a serine/threonine kinase) and parkin. In damaged mitochondria, PINK1 accumulates in the outer membrane, phosphorylates ubiquitin, and activates parkin E3 ligase activity [229,230], finally leading to mitochondria ubiquitination by parkin and degradation by autophagy [231]. Mutations in these proteins cause deficient mitophagy [232]. Furthermore, the accumulation of damaged mitochondria enhances OS and contributes to apoptotic cell death. Additional complex interactions involve α-synuclein aggregation [233]; however, the exact pathological mechanism requires further exploration.

PD pathology is also exacerbated by neuroinflammation [234]. Microglia contribute to aggregated α-synuclein clearance [235]. Upon the engulfment of α-synuclein for degradation as a protective role, microglial cells are activated; however, chronic glial activation can contribute to neurodegeneration progression in PD [236]. Activated microglia are an important source of RS, and supporting this affirmation, elevated levels of iNOS and COX in the SNpc of PD patients have been observed [237]. In addition, increased levels of other toxic agents such as TNFα in the striatum, SNpc and cerebrospinal fluid [238], and inflammatory cytokines like IL-1β, IL-2, IL-4 and Il-6 [239] have also been widely described in PD patients. Activated microglia intensify neuronal death, and in turn, dying neurons and the pro-inflammatory factors released by microglia and astrocytes amplify and perpetuate the chronic neuroinflammation, creating a toxic cycle intensifying and accelerating neurodegeneration.

### 5.3. Amyotrophic Lateral Sclerosis

ALS, known as the motor neuron disease, is characterized by progressive loss of motor neurons in the spinal cord, brainstem, and motor cortex [93]. Pathologically, the disease is characterized by the presence of cytoplasmic inclusions in degenerating neurons [240] accompanied by astrogliosis [241]. The cause of the disease largely remains unknown; in familial ALS (~10% of the cases), 20% result from mutations in SOD1 [242]. Evidence indicates that mutant SOD1 does not lose its function; in contrast, a gain of toxic function has been associated with ALS, e.g., mutant SOD1 increases NOX2-dependent RS production reducing cell survival [107]. Mechanisms leading to neuronal degeneration in ALS are complex; protein aggregation, OS, mitochondrial dysfunction, neuroinflammation, and excitotoxicity have been described [243].

Focusing on OS, there is large evidence of increased OS in ALS pathogenesis. Increased protein carbonyl levels have been detected in spinal cords of sporadic cases [244], together with increased levels of 3-nitrotyrosine residues in spinal cords of sporadic and familial forms [245,246] and CSF of patients with sporadic ALS [247]. In addition, augmented levels of 8-hydroxy-2′-deoxyguanosine (oxidative damage to DNA) [248] and HNE [249] were detected in ALS patients. Metal-catalyzed OS have been also linked to ALS pathogenesis [250]. The OS deleterious effect in ALS can be detrimental due to its interactions with other pathological pathways, including Ca^2+^ dyshomeostasis and excitotoxicity. In ALS patients, the following have been found: (i) elevated levels of excitatory amino acid present in CSF [251]; (ii) increased Ca^2+^ levels in motor nerve terminal [252]; (iii) loss of excitatory amino acid transporter 2 (EAAT2) in astrocytes, leading to increased extracellular glutamate concentration and neuronal excitotoxicity [253,254]; (iv) motor neurons that have scarce Ca^2+^ buffer systems and a high number of AMPA Ca^2+^-permeable receptors [255]. Altogether, these factors support the hypothesis that Ca^2+^-dependent excitotoxicity plays a central role in ALS pathogenesis [256]. Increased Ca^2+^ levels are buffered by mitochondria; however, during excitotoxicity, mitochondrial Ca^2+^ overload induces OS and cell death [257]. In turn, RS are able to trigger excitotoxicity [258], e.g., HNE in the spinal cords of ALS patients was responsible for induced damage to EEAT2 in astrocytes, causing excitotoxic motor neuron death [259].

ALS is also characterized by the presence of protein inclusions, and OS damage can contribute to protein misfolding and aggregation. TDP-43 was identified as a major component of ubiquitin positive inclusions in spinal cords of familial and sporadic ALS patients [260,261]; interestingly, they were not found in SOD1-familial cases [262]. TDP-43 localization is predominantly nuclear; conversely, in ALS, aggregates are located in the cytoplasm, leading to the hypothesis that cytoplasmic TDP-43 localization is toxic and induces cell death [263]. Pathological protein inclusions have been observed to be composed by other proteins, including SOD1 [264] and FUS (Fused in Sarcoma) [265]; nevertheless, these hallmarks are associated with gene mutations. TDP-43 and FUS are RNA-binding proteins, and they are rich in residues that can adapt an unfolded or aggregated state [240]; as such, aggregation in ALS can be related to a prion-like spreading of the disease [266], where one aggregated protein can sequester unfolded proteins to adopt the aggregated form. Likewise, the alteration of protein degradation pathways contributes to ALS pathophysiology. Ubiquitin presence exemplifies the involvement of UPS in protein aggregation [267,268]; indeed, proteasome inhibition in cellular models leads to increased TDP-43 levels [269]. Familial cases linked to UBQLN2 (ubiquilin 2) mutations provide another example of protein recycling dysfunction in ALS [270]. Also, different studies have reported autophagy dysregulation in motor neurons as a critical event in its pathophysiology [271,272].

Inflammation has also been related to ALS pathogenesis. Compelling evidence has demonstrated that levels of the pro-inflammatory cytokine IL-17A were increased in the spinal cords of sporadic and familial ALS cases [273], as well as levels of MCP-1 chemokine in the CSF [274]. Additional studies suggest a role for TLR/RAGE (receptor for advanced glycation end-products) signaling in ALS, e.g., increased expression of TLR2, TLR4, and RAGE was found in the spinal cords of ALS patients [275]; however, it is unclear to which extent neuroinflammation contributes to neurodegeneration. Degenerating motor neurons in ALS are enwrapped by astrogliosis. During the disease progression, astrocytes become chronically activated, they lose their neuroprotective role, and they release pro-inflammatory factors and provide a neurotoxic environment [276]. Changes in astrocyte function provide a link to the hypothesis of glutamate excitotoxicity in neuronal demise [241]. The ionotropic P2X7 receptor has been associated with neuroinflammation in ALS. P2X7 receptors are expressed in CNS cells [277], where they get activated by ATP to activate the NLRP3 inflammasome, exacerbating the microglial response and contributing to the pathogenesis of ALS [278].

## 6. Nrf2-ARE Pathway as Therapeutic Strategy

In order to prevent oxidative damage buildup, antioxidant therapies have long been pursued; however, given the involvement of RS in cellular signaling, as well as other potential causes, clinical studies with antioxidant therapies have failed in slowing or halting disease progression. As such, the up-regulation of endogenous antioxidant systems provides an alternative treatment option.

**Figure 5:**
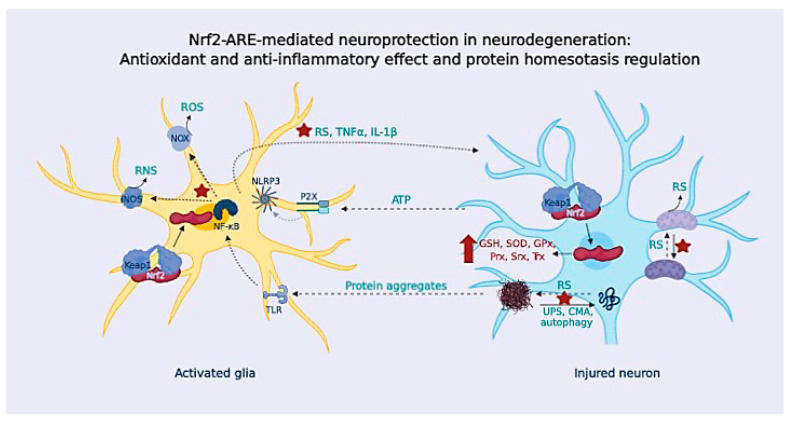
The Nrf2-ARE pathway provides neuroprotection in multiple ways. The activation of the Nrf2-ARE pathway increases the expression of antioxidant enzymes and GSH levels, thus preventing the damage to biomolecules, mitochondrial dysfunction, and neuronal apoptosis. Nrf2-ARE pathway upregulation prevents OS-driven protein misfolding and aggregation, and also stimulates the UPS and autophagy systems to control the cellular proteostasis. Additionally, the Nrf2-ARE pathway exerts anti-neuroinflammatory properties, induces the expression of the anti-inflammatory enzyme HO-1, interferes with the transcription of the inflammatory NF-κB pathway, and down-regulates NOX and iNOS-derived RS. The antioxidant and anti-neuroinflammatory effect and protein homeostasis regulation mediated by the Nrf2-ARE pathway prevents neurodegeneration.

As previously described, the antioxidant Nrf2-ARE pathway is mediated by conformational changes in Keap1, leading to Nrf2 translocation into the nucleus, and expression of the phase II response enzymes (Figure 5). While Keap1 modification mostly occurs under OS conditions, or in the presence of targeted compounds, Nrf2 can be additionally modulated by several proteins. For instance, p62/SQSTM1 competitively binds to Keap1 to release Nrf2 when it accumulates in autophagy-deficient conditions [279]. Also, as earlier discussed, GSK-3β can phosphorylate Nrf2 serine residues, creating a recognition motif for β-TrCP, an E3 ligase adapter, that mediates Nrf2 ubiquitination and proteasomal degradation in a Keap1 independent manner [184].

Nrf2, considered the master regulator of the phase II response, regulates genes involved in OS down-regulation and the upregulation of antioxidant enzymes, such as SOD, GPx, Prx, Trx, and Srx (Figure 5). In the presence of high amounts of RS, Nrf2 also regulates the expression of enzymes involved in the “*de novo*” synthesis of GSH (GCL, GR, GST) to recover GSH levels and rapidly eliminate toxic species [280]. Nrf2 also regulates several proteins related to the healthy mitochondrial function, such as the mitochondrial ETC component mitochondrial complex associated (NDUFA4), to regulate ROS production in the damaged mitochondria [281].

The Nrf2-ARE pathway is also linked to inflammatory processes. HO-1 is one of the most important antioxidant and anti-inflammatory enzymes induced by the Nrf2-ARE pathway. Moreover, Nrf2 is closely linked to the pro-inflammatory NF-κB pathway; in fact, various Nrf2 inducers have demonstrated anti-inflammatory properties via inhibition of the NF-κB pathway [282]. Several connections among the two pathways have been described, evidencing the finely balanced equilibrium; Keap1 inhibits IKKβ (the inhibitor of nuclear factor kappa-B kinase subunit beta) phosphorylation, thus blocking NF-κB translocation to the nucleus, and down-regulates TNFα [283]. In turn, NF-κB can repress Nrf2 signaling at the transcriptional level, as both factors compete for transcription co-activator binding protein (CREB binding protein, CBP) [284]. Also, NF-κB p65/relA subunit co-imports Keap1 to the nuclei to bind Nrf2 and promote its exportation to the cytosol and further degradation [285].

Additionally, the Nrf2-ARE pathway is important for cellular proteostasis. Autophagy adaptor proteins p62 and NDP52 are regulated by the Nrf2-ARE pathway [286,287]. Interestingly, they possess ubiquitin and LC3 binding sequences; thus, they are able to recruit ubiquitinated autophagy substrates to the autophagosome. Additional genes involved in cargo recognition, autophagosome formation, and autolysosome clearance are regulated by Nrf2 [288]. Likewise, Nrf2 regulates CMA through the modulation of LAMP-2A levels [289].

Thus, considering the large number of genes regulated by the phase II antioxidant response, the development of Nrf2 inducers is a highly interesting strategy in NDDs drug development. Compared to other antioxidant strategies, Nrf2 induction can regulate RS produced by mitochondria, cellular proteostasis, and neuroinflammation at the same time, recovering homeostasis. However, Nrf2 induction must be tightly regulated given that too potent an induction might be deleterious for cells, inducing toxicity and finally cellular death. Nonetheless, controlled chronic Nrf2 induction has already been demonstrated to be clinically efficacious in multiple sclerosis (MS), and it is under evaluation for other OS related diseases.

The Nrf2-ARE pathway is known to be dysregulated in several NDDs. From the above-outlined properties, Nrf2 induction has been considered a valuable strategy for developing drug candidates for the treatment of NDDs [290], as it can contribute to reducing OS, neuroinflammation, and protein aggregates present in these diseases [291,292]. Thus, targeting all these processes at the same time with an Nrf2 inducer may have a greater impact on the complex pathophysiology of NNDs. Given that the Nrf2-ARE pathway might be activated by different mechanisms, it raises the possibility to modulate the pathway via a diverse range of chemical compounds. Nrf2 up-regulation can be achieved either by direct Nrf2-Keap1 targeting or indirect modulation of connected pathways [293]. Regarding direct modulation of Nrf2-Keap1, diverse chemical compounds have been described as Nrf2 inducers, most of them being electrophilic compounds that can modify sulfhydryl groups on Keap1-cystein residues, while recent advances in the field have led to the development of protein–protein interaction inhibitors of the Nrf2-Keap1 interaction [294].

The interest of the Nrf2-ARE pathway as a suitable target to treat OS-related diseases has been demonstrated with the approval of the first Nrf2 inducer for the treatment of MS, dimethyl fumarate (DMF), in 2013 [295]. During the last years, novel structures have been developed aiming to obtain specific properties for the treatment of different complex diseases, including AD, PD, and ALS [291].

DMF was the first Nrf2 inducer approved for clinical application. It is an electrophilic compound that covalently binds to Cys residues at Keap1. DMF has been extensively evaluated as a repurposing treatment for AD, PD, and ALS with interesting results. Considering AD, DMF has demonstrated the ability to reduce neuroinflammation and improved the memory deficit in an in vivo model of neuroinflammation [296], decreasing cytokines’ expression by inhibiting NF-κB nuclear translocation. Recently, Cuadrado et al. [297] have developed a new AD transgenic model combining the expression of hAPP and hTau proteins in wild type and Nrf2 knock-out mice. In this model, DMF reduced chronic neuroinflammation, augmented autophagy-related genes, and more importantly, recovered memory function in an Nrf2 activation-dependent mode. Thus, it was demonstrated that Nrf2 activation by DMF reduces inflammation and autophagy failure triggered by hAPP and hTau expression.

DMF has also been evaluated in several PD in vivo models reproducing the main pathological hallmarks of the human synucleinopathy [298], where DMF administration reduced the loss of nigrostriatal dopaminergic neurons by decreasing the neuroinflammatory status. It reduced microglia response to α-synuclein, shifting it to an anti-inflammatory phenotype. Regarding the proteostasis system, DMF increased p62 protein levels and the LC3-II form, reducing autophagy vesicles. More importantly, DMF reversed motor deficit in α-synuclein-expressing mice in a Nrf2-dependent manner.

Considering ALS, we have previously described the implication of OS, neuroinflammation, and proteostasis in this disease. DMF related Nrf2 induction has been associated with an anti-inflammatory response [299] in autoimmune diseases. As such, DMF has demonstrated the capacity to induce the activity of natural killer cells to target dendritic cells (2, 48). Furthermore, DMF induces the anti-inflammatory phenotype, augmenting the production of IL-10, a cytokine that induces the differentiation of CD4+ T cells to a suppressive Treg subtype (2, 24). Regulatory T cells are considered a key factor to regulate ALS progression as there is a close correlation between its advance and Treg levels [300]. Thus, the immunosuppression capacity of Tregs is critical to reducing disease progression, as demonstrated by the autologous infusion of normal suppressive function Tregs that slowed ALS progression in patients [301]. Interestingly, DMF has demonstrated effective improvement of Treg levels in patients [302] augmenting the ratio of anti-inflammatory Tcell populations [303], showing a strong reduction of pro-inflammatory CD4+ INFγ+ and CD8+ INFγ+ cells. Based on observations, Vucic et al. are developing the Phase II clinical trial (TEALS Study) to evaluate the efficacy and safety of DMF in ALS patients [304].

## 7. Limitations and Future Perspective of the Antioxidant Therapy

OS is considered an important component in the development of NDDs. Thus, it has been used as a target in many clinical trials towards different OS-associated diseases, including AD, PD, and ALS, among others. However, to date, none of them has been demonstrated to be effective for the treatment of these diseases. Recently, a novel antioxidant based on spin trapping was tested, compound NXY-059, that initially showed encouraging results, but finally, it failed to demonstrate efficacy [305]. Similarly, many other well-characterized antioxidants showed disappointing clinical results, such as tirilazad [306], ebselen [307], edavarone [308], and others, a phenomenon that has been called the “antioxidant paradox” [309].

Clinical failure of antioxidants has been attributed to several reasons [310]. One reason is the poor pharmacokinetic and pharmacodynamics of the tested compounds; usually, antioxidant drugs are highly polar molecules with high molecular weights that complicate their absorption. Furthermore, these compounds are usually characterized by a quick metabolism; thus, even if they reach the bloodstream, they are quickly metabolized and eliminated, an event that explains their poor distribution and low bioavailability. These properties also complicate the crossing of the different barriers, especially the BBB, explaining part of the failure of these types of compounds in NDDs.

Another possible reason is the time and stage of administration. OS might be related to the initial stages of the disease; however, in later stages, there are many other pathological pathways involved in the advance of a particular disease. Thus, the application of antioxidant compounds might reduce the OS status without any activity in other pathological processes. In the particular case of NDDs, a general consensus is that the pathological process might start 10–15 years before the manifestation of the first symptoms; therefore, interventions at later stages might not be efficacious.

An important possibility that explains antioxidants’ failure is the poor translation between animal models of NDDs and human patients. Laboratory animals selected are usually young, healthy, and homogeneous; however, patients are usually aged persons with a high number of comorbidities and are highly diverse. These, with other potential conditions, have demonstrated that, in general, animal models of NDDs do not simulate the real disease.

Another theory indicates that supplementation with antioxidants affects the natural redox equilibrium between pro-oxidant and antioxidant species, further increasing the redox homeostasis failure in NDDs [311]. Thus, the application of antioxidants might induce a reduction of the natural antioxidant response, increasing oxidative damage when antioxidants reduce their concentrations or bioavailability [312]. Therefore, the application of high concentrations of antioxidants might have a non-expected detrimental effect. This theory might be of particular importance in the failure of long-term antioxidants treatment of chronic NDDs.

Finally, another possibility is the poor mechanistic understanding of the antioxidant effect exerted by a particular antioxidant compound. For example, in some cases, molecules such as GSH can act via post-translational modifications to mediate protective effects; however, those protecting effects can be confused with an antioxidant effect. Thus, targeting free radicals with scavengers might not be the best antioxidant approach, and targeting the pathways that regulate their concentration can probably be a more efficacious approach. In this line, the use of the Nrf2-ARE pathway might suggest an important target to be combined with other pharmacological activities in a single molecule, an approach known as the “multitarget” drug development for NDDs.

## 8. Conclusions

NDDs are among the most complex diseases, and the initial causes remain elusive. Considering the physiopathology of these diseases, there are a plethora of common pathological mechanisms involved in onset and development, such as the apparition of aberrant protein aggregates, oxidative damage, metal ions dyshomeostasis, abnormal neuroinflammatory response, and extensive neuronal death. The existence of common pathological pathways in different NDDs points to this hypothesis in which common dysregulated processes cause initial damage over time and, after crossing the no-return-point, the formation of aberrant protein aggregates contributes to the exponential increase of damage, accelerating the advancement of the disease. This hypothesis agrees with the current consensus that the onset of NDDs could occur almost twenty years before the first clinical symptoms appear. In addition, the involvement of different brain systems might be related to an increased susceptibility of a particular subset of neurons due to still debated causes.

In this review, we highlight only a small piece of the vast complexity of the pathological network underlying NDDs like AD, PD, and ALS. The existence of low grade and chronic dysregulation in several key systems for cellular survival is increasingly recognized as a potential event occurring at the very early stages of the disease. More importantly, we have pointed to a few of the multitude of interconnected pathways between oxidative stress, neuroinflammation, and proteostasis, indicating the existence of a vast number of feedback loops that augment and accelerate the damage in each particular disease. However, many questions remain unsolved and, in our opinion, the most important question to solve is in which cases this low grade and chronic dysregulation becomes pathologic and how to diagnose it.

Considering drug development programs, the design of novel and real disease-modifying drugs should be driven based on the existence of these highly interconnected pathways. Considering the evidence described in this review, we believe that there are several systems that must be taken into consideration. In particular, the key role of damaged mitochondria, the failure of mitochondrial ETC complexes in different NDDs, and the presence of high levels of oxidized products in postmortem brains of NDDs patients indicate that oxidative stress might be an early event in NDDs. In addition, it has been widely demonstrated that oxidative stress damage induces neuroinflammation and both have intensive crosstalk to finally induce a highly toxic environment for neurons. Moreover, both pathological pathways are closely interconnected to the proteostasis system, in many cases, to inhibit its activity or damage its components to create the “perfect storm” that might be the driving force during the decades before the apparition of the first clinical sign. Thus, novel drugs or treatment combinations targeting all three pathological pathways, and, even more important, the crosstalk between them, might be the path to finding an effective treatment for NDDs.
